# Mechanical evaluation of soil and artisanal bricks for quality masonry product management, Limpopo South Africa

**DOI:** 10.1038/s41598-024-64332-w

**Published:** 2024-06-17

**Authors:** George Oluwole Akintola, Francis Amponsah-Dacosta, Sphiwe Emmanuel Mhlongo, Khensani Eullen Matsiketa

**Affiliations:** Department of Earth Sciences, Faculty of Science, Engineering, and Agriculture, Private Bag X5050, Thohoyandou, 0950 Limpopo Province South Africa

**Keywords:** Soil, Artisanal bricks, Mechanical evaluation, Quality masonry, Environmental social sciences, Engineering

## Abstract

The selection of raw materials to produce quality artisanal bricks is imperative for sustainable building in rural regions. Artisanal brick-making process often employs traditional kiln to fire brick because it is an affordable, and applicable technology in the rural region. However, there are noticeable cracks, increasing among buildings constructed with artisanal bricks from the rural region in South Africa. In response, this study aims to evaluate the soil and artisanal brick specimens to understand the suitability of the raw materials and quality of products in the study area. A total of twenty soil samples and twenty-seven artisanal burnt bricks were collected from three different artisanal brick-making sites designated as Site A, B, and C. In all samples, the geotechnical tests revealed a sandy loam soil type with a predominance of chlorite clay minerals and non-clay minerals. Furthermore, the sand-size particles depict a relatively higher proportion compared to clay-size particles. Besides, Atterberg’s limit test plotted above the A-line of the plasticity chart indicates an inorganic clay of low plasticity with a low to medium compressibility property. Based on the empirical workability and mechanical tests, most of the studied soils are suitable for optimum and acceptable extrusion bricks and suitable for an on-site single-story construction based on SANS 227:2007 standards.

## Introduction

The selection of raw materials used for burnt brick in the rural region is critical to quality masonry products because artisanal clay brickmakers apply indigenous knowledge without taking into consideration the compositions of raw materials. Unlike the mechanized commercial fired brick manufacturers which employ modified clay materials with additives such as coal ash^1–3^ and geopolymers^4–6^ to improve the final clay brick quality, the artisanal brickmaker uses pristine materials. In rural regions such as Dididi village, there is an increase in cracks that develop on clayey masonry units, compromising the safety of residents and the aesthetics of structural buildings. However, several studies have reported the use of pristine soil material predominated with smectite, kaolinite, and illite as a suitable material for good-quality masonry products.

Raw material characteristic determines soil quality which, in turn, affects the quality of the final brick products. Particle sizes of raw materials have a decisive quality influence on masonry brick depending on the selected firing technology^1^ and the relative proportion of clay, silt, and sand size diameters^7^. The various size diameter has an impact on the cohesive, forming characteristics, and drying properties of the final product^8^. In practice, a raw material for brickmaking should contain a fine fraction of about 10–50% and 30% sand particles^9^. Additionally, it has been noted that a mixture of about 30% sand and 70% plastic clay is suitable quality bricks^10^. A previous study^11^ indicated that a suitable soil material for making bricks should contain no more than 60% SiO_2_, not less than 15% Al_2_O_3_, not less than 3% Fe_2_O_3_, CaO, MgO, and not less than 4% K_2_O. It has been reported that raw materials that are rich in fine particles with a diameter < 20 um and high plasticity may offer considerable prospects for the ceramic industry^12^.

The mechanical strength and durability of bricks are dependent on the mineral present in the clay material and the heat intensity to which the brick is subjected. A mineralogical composition comprising quartz, kaolinite, illite, feldspar, and smectite has been reported to characterize raw materials suitable for various red ceramic bodies at 900 °C, 1000 °C, and 1100 °C^12,13^. Temperatures of 900 °C and above cause vitrification to occur. This means that a small quantity of glass-like material forms which helps glue all the elements in the clay together. As such, the final quality of the brick depends mainly on the degree of vitrification^14^.

Despite decades of studies on fired bricks for construction material throughout the world, the quality of bricks is still a major concern, particularly, in the rural region. In the study area, previous studies^15^ have focused on modified clay materials for environmental sustainability. However, there is a paucity of studies on pristine raw materials and artisanal bricks produced. As such, this study evaluates both raw materials and artisanal bricks manufactured in the study area to understand the quality of masonry-fired brick used for construction buildings.

## Study area

**Figure 1:**
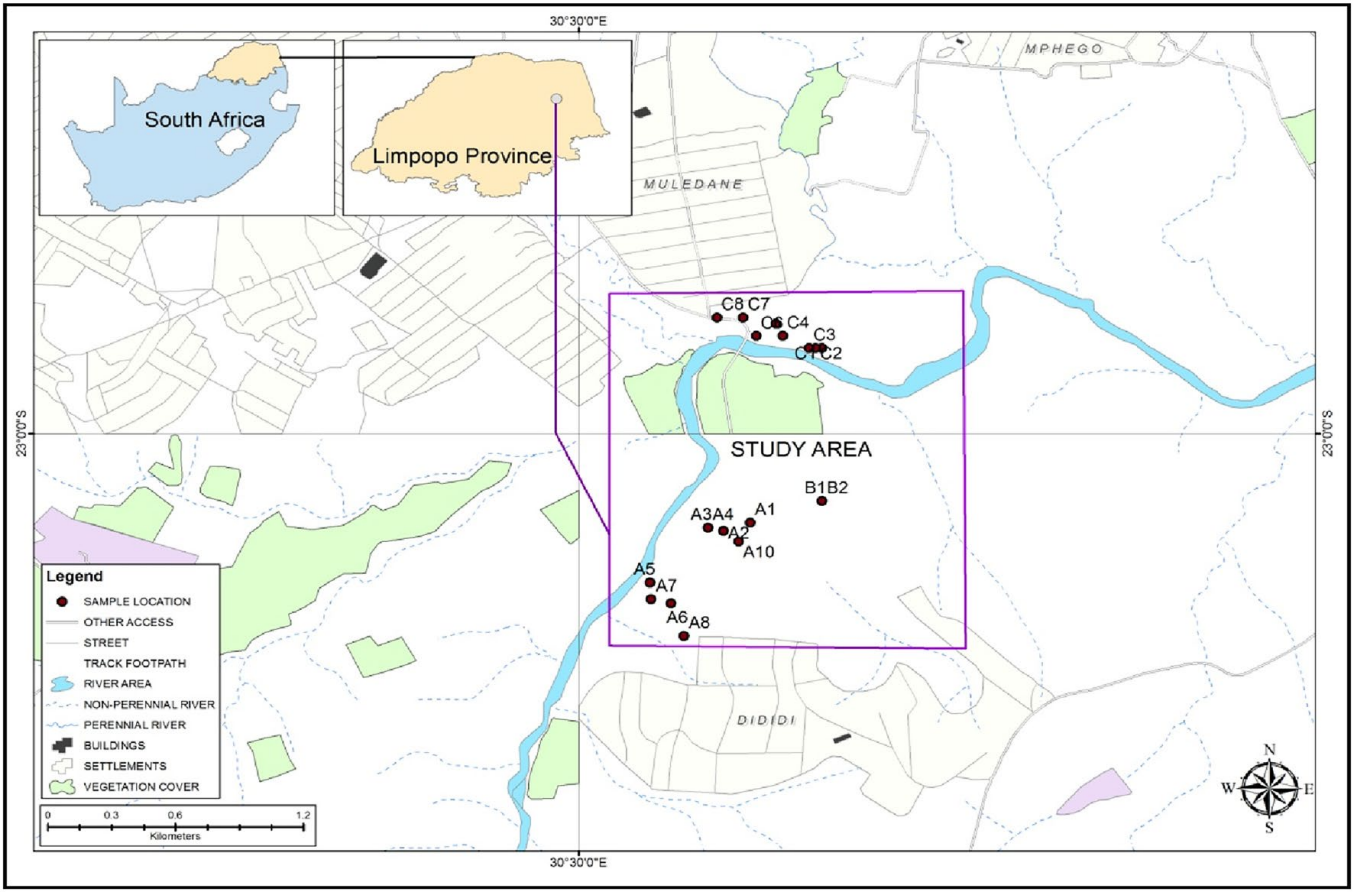
Distribution of samples in the Study Area (created with QGIS 3.22).

The study area is situated at the artisanal brick-manufacturing sites (A, B, and C), about 73 m from the Luvhuvhu River in the Dididi village of Thulamela Municipality, Limpopo Province of South Africa. The geographic coordinates of the study area are between 23°0′0″ S and 30°30′0″ E as shown in Fig. 1. The study area is underlain by the Precambrian basalts of the Soutpansberg Group^16^ and characterised by pyroclastic lava of about 3000 m thickness which intercalates sedimentary rock with tuffaceous rock^17^. However, the sediment thickness varies at different locations and has an estimated thickness of 200 m in the Mutshindudi Natal House agglomerate north of Thohoyandou but is well developed in the Kruger National Park^16^.

Chronologically, the volcano-sedimentary succession of the Soutpansberg Group comprises the basal, discontinuous Tshifhefhe Formation, Sibasa Formation, Fundudzi Formation, Willie’s Poort Formation, Musekwa Formation and Nzhelele Formation^16^. However, the Thulamela Municipality study area is mainly underlain by the Sibasa Formation which comprises mainly of arenaceous and argillaceous rocks with a few thin pyroclastic basaltic beds^16^. The volcanic and Sedimentary rocks of the Soutpansberg group occupy an East-South-East trending graben within the previously uplifted and eroded high-grade gneiss terrain of the Limpopo Mobile Belt^18^.

### Quality product management of artisanal fired bricks

**Figure 2:**
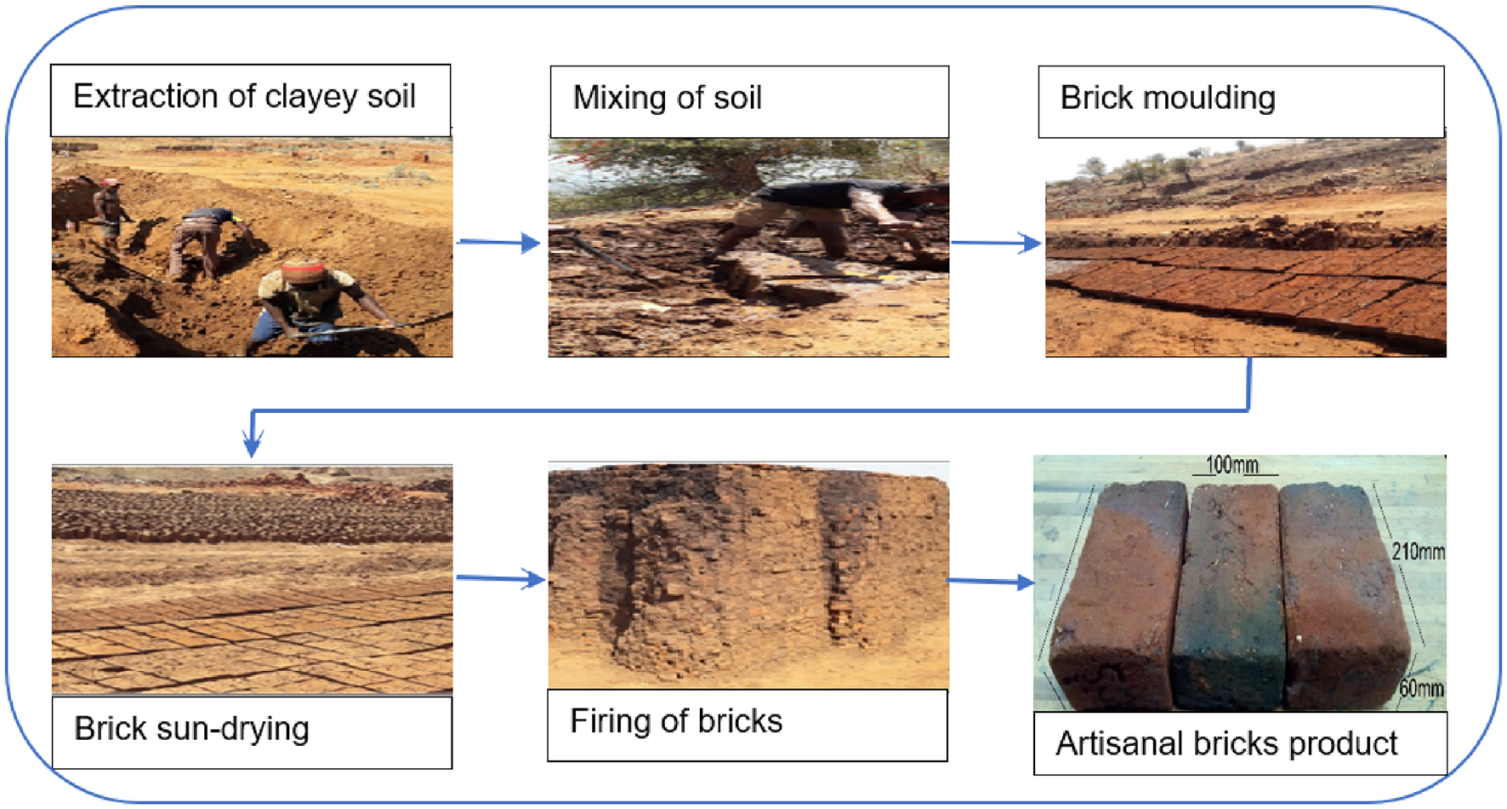
Artisanal burnt brick-making process in the Dididi studied area.

The product quality management for the artisanal brick manufacturing enterprises in Dididi village has been triggered by the issues associated with the manufacturing operations in the area. These issues were identified in the field, including but not limited to the fact that clay brick manufacturers at Dididi Village are unregistered and unrecognized by the Clay Brick Association of South Africa. The manufacturing operations employ indigenous knowledge to choose soil materials used for burnt bricks in a non-mechanised manner, following the traditional process shown in Fig. 2.

Before extraction of raw material, the area was cleared of vegetation to expose the targeted layers of clay soil, to cast their green bricks, and to subsequently sun-dry bricks before being fired. Afterward, the materials were prepared by mixing the raw materials with the right amount of water for a desired mixture for brick making. The amount of mixing water used for mud preparation differs based on the soil type because the soil from different regions has different water absorption capacities. The prepared mixture is stirred with shovels and with their bare feet. This process of mixing time takes at least half an hour. Meanwhile, from the field observations at the sites, large lumps in the materials were being reduced manually by adding water into the soil and breaking and crushing the materials using shovels. Such a traditional and manual mixing process might leave some of the material not properly reduced to the right sizes and this can, in turn, affect the quality of the final products.

Moulding was done manually using traditional hand moulding tools. As observed in the field, the mould was wet and placed on the ground, and filled with the wet soil mixture. The mould was then lifted, and the brick was left on the ground to dry. The wet bricks were covered with dry raw materials to control the moisture content as the bricks dried. Based on visual observation, the shrinkage of production mixtures varies approximately between 3 and 7%. The shrinkage amount here depends on the clay’s properties such as the mineral compositions and the particle size. The bricks were sun-dried for about 3–4 days to ensure that there was constant drying and to prevent the cracking of the bricks from overheating by the sun. However, it was observed in the field that the drying period depended mainly on the season and weather conditions. During sunny days, drying only took 2–3 days while on cloudy days it can exceed the number of days.

Upon completion of drying, the bricks were packed in stacks as shown in Fig. 2, and fired using firewood. To enhance the combustion, accessories such as macadamia nut shells, plastics, leaves, and petrol are used, and the firing process can take about 1–2 days. However, the firing temperature is generally not known as there was no temperature sensor used in all the brick firing setups. This resulted in the under-firing of some of the bricks while others were over-fired due to even distribution of heating. This was observed to be one of the factors that seriously affected the quality of bricks produced in the study area.

## Materials and methods

### Sample collection

A total of twenty soil samples and twenty-seven artisanal burnt bricks were collected from three different artisanal brick-making sites designated as Site A, B, and C at the Dididi Village. The artisanal burnt bricks were collected from surface bricks because they are considered to be better fired compared to bricks at depth. In addition, the soil samples were randomly collected in each site using a hand auger at a depth greater than 2 m to ensure homogeneity of soil particles. Meanwhile, informed consent was obtained to publish the information in Fig. 2.

### Geochemical characterisation

About 8 g of pulverised bulk soil samples were analysed for major element composition using PANalytical Axios X-Ray fluorescence (XRF) spectrometer. Samples were weighed and dried in an oven for 24 h at a temperature of less than 50 °C, then mixed with 12 g of boric acid as binder and pressed into the tin cup before being mounted on the XRF instrument. The XRF instrument equipped with a 4 kW Rhodium (Rh) tube was used to analyse major oxides by inserting a glass disk containing 1 g calcined sample, 8 g flux composed of 35% alkali borate (LiBO_2_), and 64.71% lithium tetra borate (Li_2_B_4_O_7_) as oxidant at 1050 °C into the XRF equipment. For details, previous work^12^ describes the accuracy, precisions, procedures, and standards employed for this analysis.

### Mineralogical characterisation

At the same time, the mineralogical components of the pulverised samples were obtained using X-ray diffraction (XRD) spectroscopy. PANalytical X'Pert Pro powder diffractometer equipped with X'Celerator detector coupled with receiving slits, variable divergence, and Fe-filtered Cu-Kα radiation 30 mA. Whilst the receiving slit was positioned at 0.040°, the counting area was from 5° to 70° on a 2θ scale at 1.5 s with a scan speed 1,000°/min.

### Particle size distribution

Following the ASTM standard technique (D-422-63), the particle size analysis of the soil samples was achieved by shaking approximately 500 g of the soil sample through a set of sieves of progressively smaller openings with a pan at the bottom.

### Atterberg limit determination

The degree of plasticity of studied samples was achieved through the determination of Atterberg's liquid (LL) and plastic limit (PL) using the Casagrande apparatus (Casagrande, 1947) in accordance with the ASTM-D4318 standard. Before the commencement of the procedure for liquid limit, the apparatus was properly adjusted to the consistency height of 10 mm. The correctness of calibration was confirmed by a vibrated sound when the crank struck the cam follower. Subsequent trials were repeated producing successive numbers of blows to close the grooves. The number of blows (N), as the ordinates on the logarithmic scale, was plotted against the moisture contents as the abscissae on the arithmetic scale using semi-logarithmic graph paper. The flow curve was a straight line of best fit drawn as nearly as possible through the plotted points to determine the moisture content at 25 blows.

### Mechanical tests

The mechanical tests of the artisanal burnt brick specimens encompass the determination of the compressive strength of 12 burnt brick samples and the water absorption test of 15 specimens for quality and durability at Polokwane SGS Metrolab. The bricks were weighed and recorded in grams. The brick strength was tested by determining the compressive strength of the brick against vertical load. The brick was subjected to a compressive force until failure and the failure load over the gross area was recorded as strength. The compressive strength of the bricks was tested at a loading rate of 140 kg/cm^2^ /min following international procedures ASTM C67-03 from which the national standard SANS227:2007 was adopted.

Additionally, the water absorption capacity test of the bricks was conducted to determine the durability and suitability of the bricks for building purposes using the ASTM C67-17. This test was carried out by soaking the bricks in water for a period of 24 h. A total of 15 burnt bricks (5 for each selected site) were used to carry out the test. The bricks were first weighed when dried and recorded as weight 1 (W1), then they were soaked in cold water for 24 h and were weighed again after soaking. The second weight after soaking was recorded as weight 2 (W2). The percentage of the water absorbed was then calculated. The water absorbed by the brick was indicated by the difference in W2 and W1. The water absorption value for each site was indicated by the average values of the brick replicates for each location.

## Results

### Particle size analysis of studied soils

**Figure 3:**
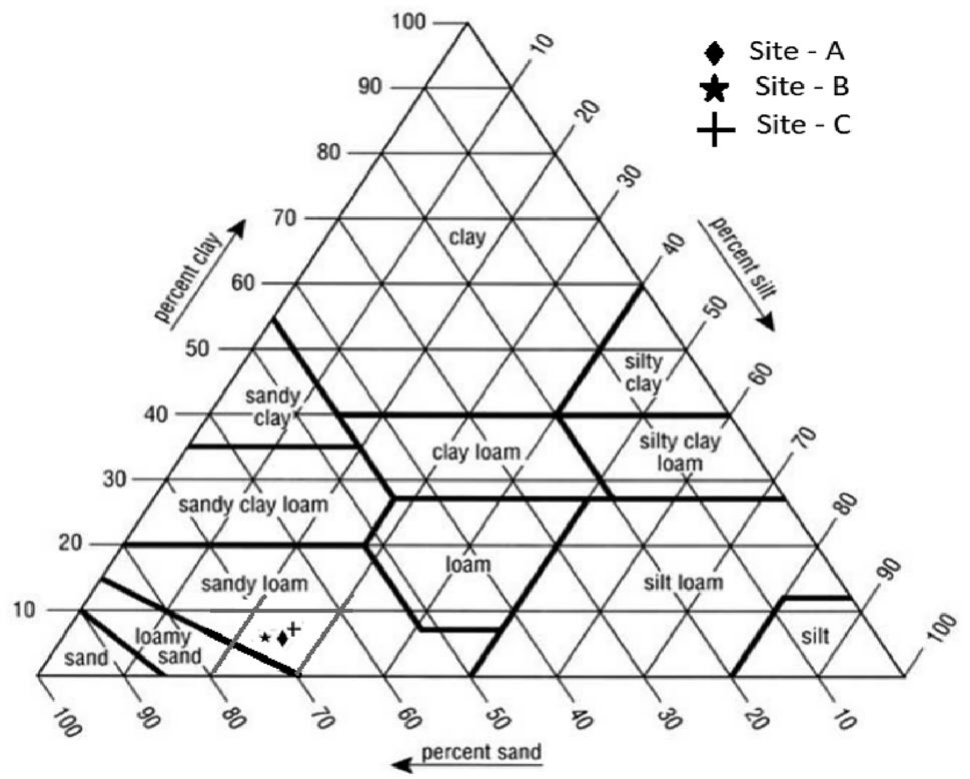
Soil texture classification of the studied samples.

Particle size analysis in all the studied sites depicts a relatively high proportion of sand size (77–79%), followed by silt (17–18%), and clay (4–5%) as shown in Table 1, belonging to a sandy loam classification. Based on the Unified Soil Classification System (USCS), the studied samples were classified as graded sand with little fines because the uniformity coefficient (C_u_) value is greater than value 1. At the same time, the C_u_ indicated that the studied soils consist of particles with different size ranges as shown in Fig. 3. In most samples, the proportion of fine particles from size diameter < 2 to 50 µm constitutes about 30% of the bulk sample. Previous studies have noted that a value of C_u_ greater than 4 to 6 classifies the soil as well-graded. When C_u_ is less than 4, it is classified as poorly graded or uniformly graded soil.

**Table 1:** Physical and chemical properties of the studied soil.

Properties	Parameters	A	B	C
Physical properties (%)	Clay (< 2 µm)	5	4	4
	Silt (2–50 µm)	18	18	17
	Sand (> 50 µm)	77	79	79
	Uniformity Coefficient (C_u_)	10	8.6	7.1
	Curvature coefficient (C_c_)	14.4	8.3	10
	Classification USCS	SW	SW	SW
Atterberg limits (%)	Liquid limit (W_L_)	29.46	25.65	30.58
	Plastic limit (W_p_)	15.22	17.13	27.22
	Plasticity Index	14.25	8.49	3.36
	Plasticity	Medium plastic	Low plastic	Slightly plastic
Chemical compositions (%)				
	SiO_2_	54.12	54.52	52.44
	Al_2_O_3_	14.20	13.89	14.45
	Fe_2_O_3_	5.80	5.79	6.17
	Na_2_O	0.85	0.67	0.71
	K_2_O	2.68	0.67	0.71
	MgO	1.70	1.91	1.71
	CaO	1.54	1.61	1.44
	MnO	0.11	0.92	0.80
	TiO_2_	1.24	1.34	1.33
	P_2_O_5_	0.16	0.18	0.15
	LOI	17.6	18.5	20.1
	SiO_2_/ Al_2_O_3_	3.83	3.93	3.63

SW indicates well graded sands, gravelly sands with little or no fines.

It has been reported^19^ that an ideal grain size distribution of the soil to achieve good quality bricks should have about 20–40% sand, 25–45% silt, and 20–35% clay-sized particles. Retrospective study In the same line, a previous study^20^ has suggested that the suitable material for making clay bricks should contain about 30% of sand size particles as it reduces shrinkage that mostly occurs during the firing of soft clayey material. At the same time, previous work^21^ has indicated that soil for brickmaking should contain about 10–50% of clay fraction and 30% of sand size particles. According to Fernandes^10^ prehistoric brickmakers often used a clay mixture of about 30% sand and 70% plastic clay. In the study area at Dididi, the particle size distribution shows a dispersion from recommended values since it has sand particles ranging from 77 to 79%, silt ranging from 17 to 18%, and clay ranging from 4 to 5%.

### Atterberg limit test of studied soils

**Figure 4:**
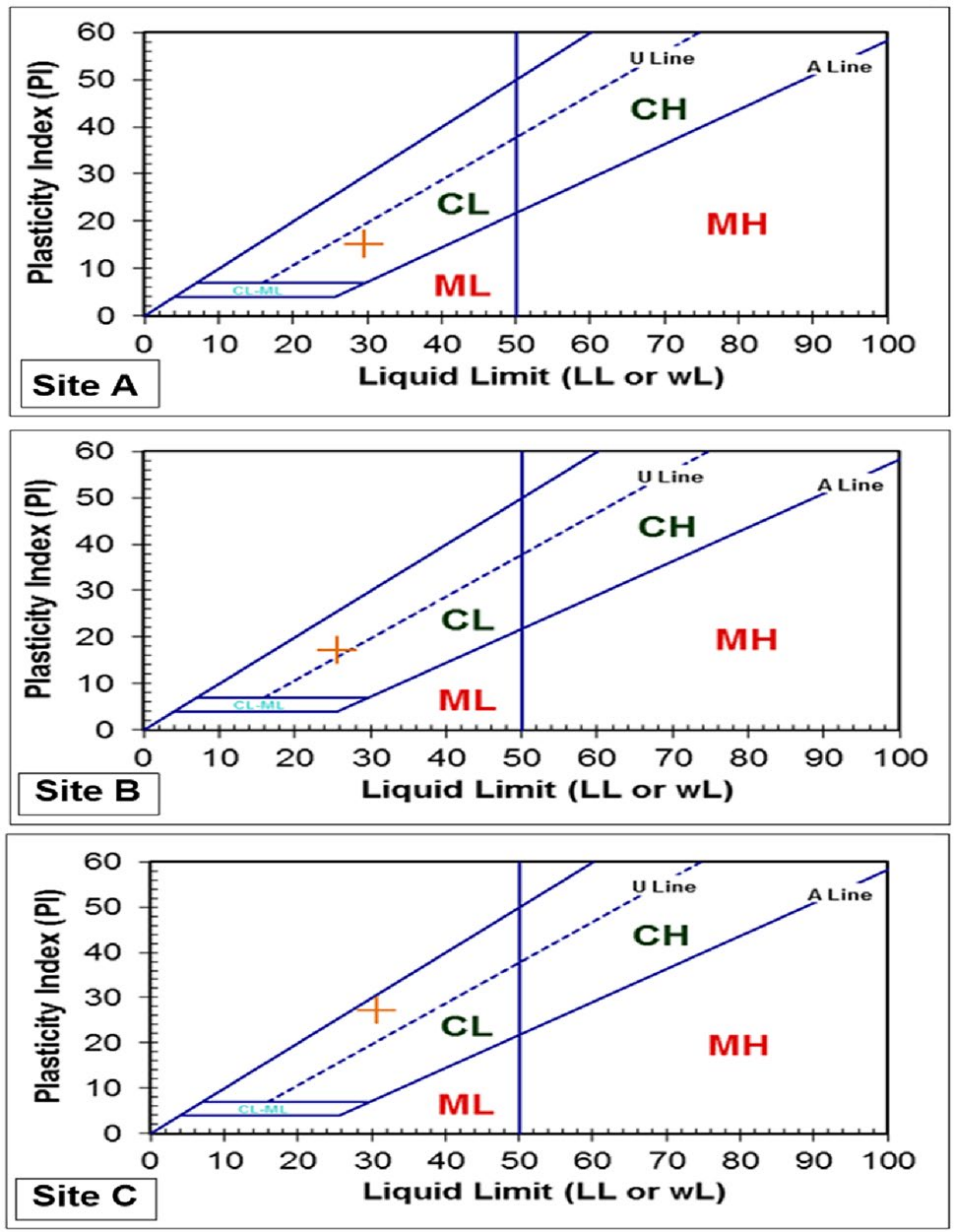
Atterberg’s limit determination.

Plasticity limits in all the studied sites varied from 3.36 to 14.25% (Table 1). Most of the studied samples plotted above the A-line (Fig. 4), indicating an inorganic clay of low plasticity with a low to medium compressibility property while site C plotted above the U-line, showing a cohesionless soil. The Atterberg limit tests showed the highest value of liquid limit (30.58%) and plastic limit (27.22%) at site C compared to sites A and B. At the same time, site C exhibits the lowest plasticity index value (3.36%) compared to site A (14.25%) and site B (8.49%) probably due to the high content of silica. Based on the Casagrande chart, the studied samples collected from site A belong to the domain of medium plasticity while sites B and C belong to a low plastic domain.

Regarding brick-making soil, studies have indicated that the minimum expected plastic index value should range from 7 to 16%^14^, a liquid limit value ranging from 25 to 47.7%^22,23^, plasticity index values ranging from 7 to 24.14%^24^ are required in making high-quality clay bricks. Considering the liquid limit and plasticity index values presented in Table 1, the soil used for manufacturing fired bricks at Dididi possesses a range of plasticity from low to medium index, depending on the location in which the soil was collected.

Soil collected from Site A and Site B was found with plasticity values falling within the threshold recommended in some previous studies^22,23^. Soil from Site C was characterised by less plasticity value compared to Site A and B, and it can be inferred that soil from Site C does not have adequate plasticity required for moulding the bricks into good quality shaped bricks.

### Geochemical characterisation of studied soils

The major oxides analysis reveals the presence of SiO_2_, Al_2_O_3_, Fe_2_O_3_, Na_2_O, K_2_O, MgO, CaO, MnO, TiO_2_, and P_2_O_5_ oxides as shown in Table 1. In all studied samples, the predominant oxide is SiO_2_ (52.44–54.52%), followed by Al_2_O_3_ (13.89–14.45%), and Fe_2_O_3_ (5.79–6.17%), while other oxides show an average of less than < 1.0%. The ratio of SiO_2_/ Al_2_O_3_ is > 3.5 and ranged from 3.63 to 3.93, indicating excess silica content which could act as filler material, decreasing firing shrinkage in all the studied samples. The low proportion of fluxing oxides comprising Na_2_O, K_2_O, CaO, and MgO (< 2%) may cause insufficient sintering during firing, thus lowering the densification of the final product. The relatively low proportion of TiO_2_ and P_2_O_5_ (< 2%) may favour the dark coloration of all the samples.

It has reported the main constituents of clay bricks with proportions of silica ranging from 53 to 61% and alumina varying from 22 to 32%^10^. According to the previous study^11^ normal clay for making bricks should contain not less than 60% SiO_2_, not less than 15% Al_2_O_3_, not less than 3% Fe_2_O_3_, CaO, MgO, and not less than 4% K_2_O. it is important to note that a good clay material for brickmaking contains SiO_2_ ranging from 50 to 60%, Al_2_O_3_ ranging from 20 to 30%, CaO 10%, less than 7% Fe_2_O_3,_ and 10% alkalis^25^. In addition^26^, previous studies have reported the chemical analysis results of good soil for making clay bricks as follows; SiO_2_ (61.8%), Fe_2_O_3_ (> 3%), and Al_2_O_3_ (23%).

The chemical composition of the studied samples is pozzolanic materials based on the summation of the percentage of the oxides of silicon, iron, and aluminum being greater than 70%^27^. The amounts of silica and titanium oxide aligned with some of the previous findings^28,29^. However, the values obtained for alumina are relatively lower than the recommended values^25,29^. Alumina gives the plasticity needed for moulding the bricks into the required shape; therefore, an inadequate quantity of alumina in the soil lowers the plasticity of the soil, resulting in the production of bricks with poor bond^30^.

Excess amount of silica in the soil for brickmaking decreases the cohesion property and renders the bricks brittle. Although the fluxing oxides were present in a relatively low proportion, their presence in the raw materials used to manufacture clay bricks has a great influence on the final properties of the bricks. The fluxing elements in the brick-making soil are necessary to enable the silica to melt and bind the particles during the firing^11,31^. However, the elements may also cause insufficient sintering and bloating during the firing stage. Excessive amounts of fluxing elements such as sodium oxides (Na_2_O) and potassium oxide (K_2_O) in the soil used for brick manufacturing may cause the bricks to crack and warp. The presence of CaO in the soil may change the interior colour of the brick while the titanium oxide influences the overall hue of the bricks.

### Mineralogical characterisation of studied soils

**Figure 5:**
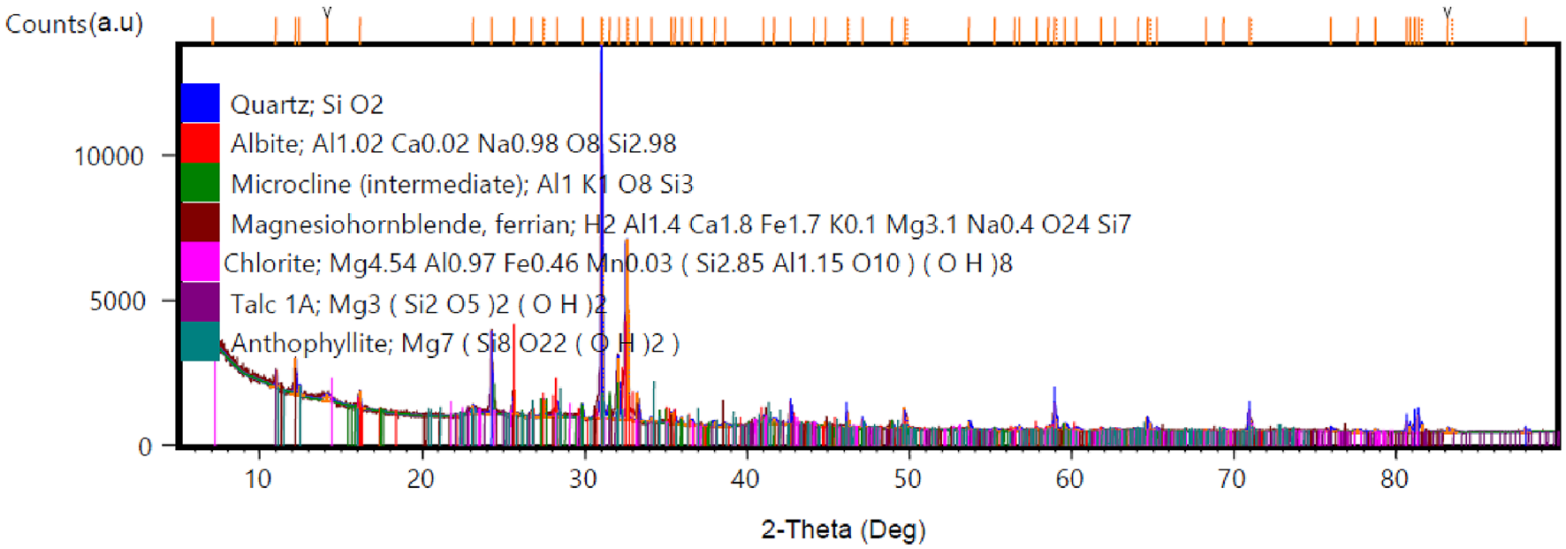
Representative XRD patterns of the studied soil.

The XRD diffraction of studied soil samples is presented in Table 2. The main clay minerals were chlorite and talc while the non-clay minerals comprised quartz, plagioclase, microcline, hornblende, and anthophyllite (Fig. 5). The preponderance of quartz mineral (34.16–36.99%) may reduce the plasticity properties of the clay mineral chlorite (7.90–8.50%) and talc (6.82–7.05%) since it is brittle and cohesionless. The varied content of chlorite in the studied site A (7.90%), site B (7.90%), and site C (8.50%) may be attributed to different detritus magnesian enrichment from metamorphosed basaltic parent rock. To corroborate the provenance source of the studied sample, the presence of hornblende minerals (5.09–5.80%) and anthophyllite (0.79–3.88%) an amphibole mineral suggests the derivation of sediments from a low-grade metamorphosed parent rock. Unlike smectite clay minerals, the talc and chlorite minerals are characterised by a non-swelling and low plasticity index. The presence of a high relative proportion of plagioclase (28.57–32.09%) is expected as typical of alluvial sediments from basaltic source rock which can act as a flux and lower vitrification temperature. While the mineralogical composition in this present study varied widely from previous work^15^, the presence of pyrophyllite, a talc mineral, and non-clay minerals suggest similar provenance from parent material. The absence of clay minerals such as kaolinite, vermiculite, and smectite in the present study, which was detected in previous work conducted in Dididi indicated a different degree of weathering at Dididi village.

**Table 2:** Mineralogical composition of the studied sites.

Sample	Mineral composition (%)						
	Anthophyllite	Chlorite	Hornblende	Microcline	Plagioclase	Quartz	Talc
Site A							
A1	1.38	6.12	5.98	11.21	29.66	39.12	6.54
A2	–	8.42	3.96	15.01	22.78	43.03	6.8
A3		7.42	6.46	9.34	33.57	38.53	4.68
A4	2.99	7.76	6.1	13.31	28.26	33.03	8.55
A5	1.57	10.59	4.61	11.67	29.42	35.81	6.32
A6	3.72	5.26	7.22	10.85	29.45	35.12	8.37
A7	–	9.69	4.66	12.43	28.91	37.63	6.68
A8	–	5.87	2.37	13.79	35.08	37.58	5.3
A9	–	11.81	4.58	12.62	34.42	29.27	7.3
A10	3.88	6.01	4.97	10.09	28.43	33.46	7.66
Average	2.71	7.90	5.09	12.03	30.00	36.26	6.82
Site B							
DID-11		8.82	5.76	13.36	29.06	37.5	5.5
DID-12	2.26	5.81	4.96	12.76	35.11	30.81	8.29
Average	2.26	7.90	5.36	13.06	32.09	34.16	6.90
Site C							
DID-13	1.46	5.98	7.06	12.09	31.57	35.33	6.51
DID-14	–	7.36	6.12	12.02	33.54	37.18	3.78
DID-15	–	11.3	4.98	13.78	25.46	36.15	8.34
DID-16	–	13.2	6.35	10.97	21.6	37.96	9.91
DID-17	0.92	1.11	4.93	15.39	29.79	42.24	5.62
DID-18	–	13.02	6.58	11.92	25.36	34.89	8.24
DID-19	0.79	7.23	4.99	13.58	29.29	37.17	6.94
DID-20	1.25	8.8	5.41	10.51	31.94	35.01	7.07
Average	1.11	8.50	5.80	12.53	28.57	36.99	7.05

### Compressive strength test of the burnt bricks

The compressive strength of the artisanal bricks and the required South African National Standards (SANS 227: 2007) for quality burnt bricks are presented in Table 3. Based on the results, Soil C is slightly plastic, while soil A and B exhibit medium and low plasticity respectively. However, the compressive strength value of Soil C (3.8MPa) is higher than Soil B (2.8 MPa) but similar value with Soil A (3.8 MPa). The plausible explanation could be attributed to higher amounts of chlorite clay mineral, in soil C, which provided adequate strength for the final brick^32^. The compressive strength of bricks produced in Site A and C (3.8 MPa) tends to meet the SANS standard than bricks produced at Site B (2.9 MPa). It suggests that the artisanal bricks collected from Site A and C showed suitability for an on-site single-storey construction for individual bricks. An on-site manufacturer is not required to be transported more than 25 m to the place where they will be built into the structure. On the contrary, the average compressive strength values obtained in this study show that the artisanal brick does not meet the required average compressive strength for burnt clay bricks. The SANS standard stipulates the average compressive strength values of 4.0 MPa for on-site manufacture, 5.0 MPa for off-site manufacture, and 10 MPa for double-storey constructions.

**Table 3:** Comparison of the compressive strength test of the artisanal bricks with standards.

	Brick making		
	Site A	Site B	Site C
Mass (g)	4245.5	4282.25	3649
Average strength (MPa)	3.8	2.9	3.8
Required compressive strengths of burnt bricks (SANS 227:2007)			
Description	Average		Individual
Single-storey construction:			
On-site manufacture	4.0		3.2
Off-site manufacture	5.0		4.0
Double-storey construction	10.0		8.0

On-site manufacture is where units do not require to be transported more than 25 m to the place where they will be built into walls.

### Water absorption property of the burnt bricks

Table 4 presents the results of water absorption tests carried out on the artisanal bricks collected from the three selected brick manufacturing sites in Dididi. Fifteen burnt bricks comprising five from each site, were tested for water absorption property. The results revealed the average water absorption values of 13.5, 15.0, and 16.1% for Site A, B, and C respectively. Samples from Site B and C were noted to have a relatively higher absorbent property than brick samples from Site A. The results showed that bricks in Site A fall below the requirements of SANS 227:2007 of less than 14% accepted water absorption value. Bricks from Site B and C exhibited higher values of water absorption and did not dissolve in water whereas a sample, A4, from Site A did dissolve completely in water within a period of 24 h. This could be attributed to the under-firing of these bricks during the firing process. The under-fired brick can absorb water to expand and reduce repeatedly when it rains or freezes, resulting in cracks in the buildings.

**Table 4:** Water Absorption Capacity of the Fired Bricks from the Study Area.

Sample number	Initial weight of dry bricks W1 (kg)	Final weight of bricks W2 (kg)	Water absorption $\frac{w2 - w1}{{w1}} \times 100$	Average % water absorption
Site A				
A1	2.619	2.993	14.3	13.5
A2	2.562	2.905	13.4	
A3	2.526	2.854	13.0	
A4	2.516	–	–	
A5	2.713	3.074	13.3	
Site B				
B1	3.762	4.332	15.2	15.0
B2	3.993	4.551	14.0	
B3	3.479	3.988	14.6	
B4	3.432	3.963	15.5	
B5	3.964	4.580	15.5	
Site C				
C1	3.140	3.643	16.1	16.1
C2	3.040	3.567	17.3	
C3	3.335	3.911	17.3	
C4	3.098	3.644	17.6	
C5	2.896	3.406	17.6	

“– “ indicates the brick dissolved in water.

## Discussion

The interplay of properties including mineralogical, geochemical, physical, and mechanical characteristics of studied soil materials contribute to artisanal brick quality. The presence of chlorite, talc, and anthophyllite minerals suggests a provenance source of studied soil from a low metamorphic parent material. The absence of a high plastic mineral (PI > 35%) such as smectite or kaolinite mineral might have lowered the plasticity of the studied soil, thus chlorite and talc minerals apparently imparted low to moderate plasticity properties on the soil. In the same vein, an alluvial soil collected from a parent material composed of chloritic shist has shown a medium to high plasticity in Ayos alluvial soil^33^. Unlike the Dididi alluvial soils, the Ayos alluvial soils contain kaolinites indicating a high degree of weather of the chloritic shist parent rock. The Dididi alluvial soil occurs in a low weathered soil, relatively low stable acidic environment, with incomplete weathering to produce smectites and vermiculites minerals^34^. Additionally, alkali and earth alkaline minerals such as K_2_O, Na_2_O, CaO, and MgO are considered good fluxing agents in brick manufacturing, reducing melt temperature, and contributing to the rapid vitrification behaviour of the final brick.

**Figure 6:**
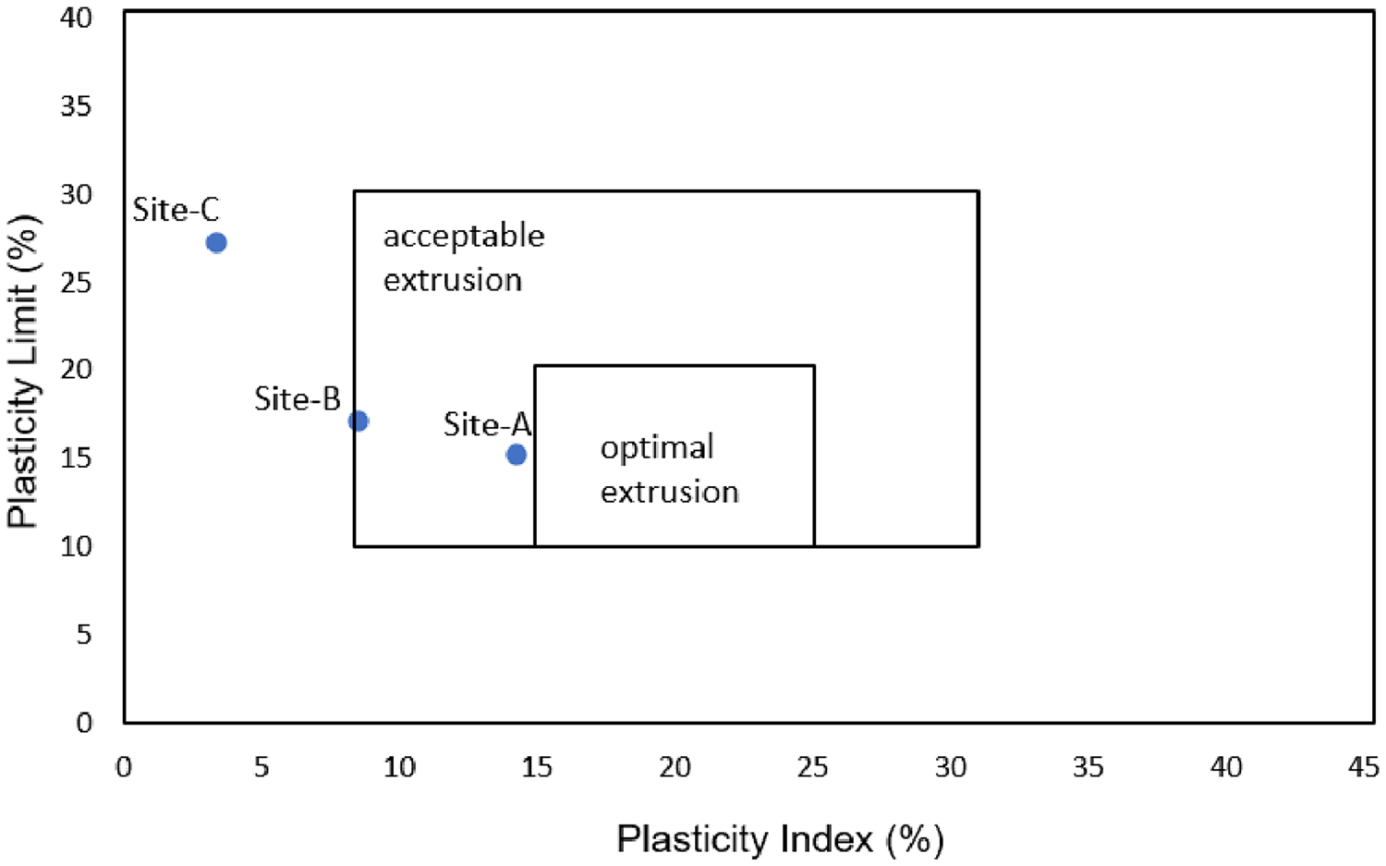
Diagram showing optimal (**A**) and acceptable (**B**) domains for clay shaping by extrusion^41^.

The plasticity of the studied sites is favourable for acceptable—extrusion and manual workability due to the presence of the chlorite and talc minerals based on the extrusion analysis chart (Fig. 6). The presence of chlorite and talc minerals in the soil used for brick manufacturing plays a significant role in facilitating the molding properties of the soil^35^. With respect to quality, chloritic soils can make quality bricks as they produce lightweight aggregates when fired rapidly under reducing conditions^36^. Apart from talc mineral capable of increasing the vitrification of the material, it can be used as a flux to reduce the expansion of bricks provided it is present in low quantities^37,38^. Furthermore, the high amount of quartz content found in the studied samples revealed the predominance of silica (SiO_2_) in the samples which can act as a filler material, decreasing the firing shrinkage of the soil^30,39^. Additionally, the high proportion of quartz content together with metal oxides in brick-making soil increases vitrification temperature and reduces drying and firing shrinkage^40^. The raw materials used to manufacture clay bricks in Dididi are dominantly composed of chlorite clay mineral and quartz non-clay materials which may contribute to the good quality of the final bricks.

The compressive strength test of the brick relates to the durability and load-bearing capacity of the bricks under maximum axial compressive stress. In this present study, the artisanal burnt bricks collected from Dididi exhibited a low compressive strength compared to the artisanal bricks evaluated by previous work^15^ due to mineralogical and particle distribution heterogeneity. The moderate proportion of highly plastic clay minerals, having PI < 35%, reduces the dimensional crushing of bricks during compressive strength test^42^. At the same time, the durability of bricks depends on the proper proportion of quartz in the sample^43^.

The water absorption capacity of the artisanal bricks is a function of the dominant particle sizes, mineral compositions, and the firing temperature. Generally, the dominance of coarser grains with a diameter > 50 µm intrinsically increases the pores of moulded brick to absorb water^28^. Additionally, the glassy phase resulting from firing bricks in the kiln tends to fill the pores present in the artisanal bricks, decreasing the porosity level of the burnt bricks. Alluvial clay deposits have a water absorption capacity lower than 25% which alludes to the ASTM 34-13 standard threshold^28^. The water absorption capacity (13.5–16.1%) of this present study corroborated with the previous finding on WA (9.8–6.5%) for the fired bricks from Dididi^15^ but there is a disparity in the compressive strength values. This disparity could be attributed to the heterogeneous distribution of heat during the Kiln firing of moulded bricks.

## Conclusions

Artisanal burnt bricks used for building constructions at the study area were categorised as sandy loam, ranging from low to medium plasticity, predominated with chlorite clay mineral and talc in a relatively low proportion compared with quartz and feldspar non-clay minerals. Based on the empirical workability chart, most of the studied soils are suitable for optimum and acceptable extrusion bricks. On the other hand, based on the mechanical tests, all the studied brick samples were found to have water absorption capacity within acceptable standards while the compressive strength of brick samples from sites A and C are suitable for an on-site single storey construction for individual bricks due to heterogeneous distribution of heat during kiln firing of moulded bricks. The artisanal brick-making process lacked a modern firing temperature regulator to control the burnt brick during Kiln firing.

## Data Availability

The datasets generated and/or analysed during the current study are available in the UnivenIR repository, https://univendspace.univen.ac.za/handle/11602/1129.
